# Conceptual design of the Hybrid Ring with superconducting linac

**DOI:** 10.1107/S1600577521012753

**Published:** 2022-01-01

**Authors:** Kentaro Harada, Nobumasa Funamori, Naoto Yamamoto, Yoshito Shimosaki, Miho Shimada, Tsukasa Miyajima, Kensei Umemori, Hiroshi Sakai, Norio Nakamura, Shogo Sakanaka, Yukinori Kobayashi, Tohru Honda, Shunsuke Nozawa, Hironori Nakao, Yasuhiro Niwa, Daisuke Wakabayashi, Kenta Amemiya, Noriyuki Igarashi

**Affiliations:** a Accelerator Laboratory, High Energy Accelerator Research Organization (KEK), Tsukuba, Ibaraki 305-0801, Japan; b Institute of Materials Structure Science, High Energy Accelerator Research Organization (KEK), Tsukuba, Ibaraki 305-0801, Japan

**Keywords:** light source, ultrashort pulse, ultralow emittance, two-beam application

## Abstract

The Hybrid Ring with a superconducting-linac injector as a highly flexible synchrotron radiation source to enable new experimental techniques and enhance many existing ones is proposed. It is designed to be operated with the coexistence of the storage (SR) bunches characterized by the performance of the storage ring, and the single-pass (SP) bunches characterized by the performance of the superconducting linac. Unique experiments can be performed by simultaneous use of the SR and SP beams, in addition to research with various experimental techniques utilizing the versatile SR beam and research in the field of ultrafast dynamics utilizing the ultrashort pulse of the SP beam. The extendability of the Hybrid Ring will allow it to be developed into a synchrotron radiation complex.

## Abstract 2

The concept of the Hybrid Ring, designed to be operated with the coexistence of two types of beams, is presented, with examples of application.

## 1. History of synchrotron radiation sources and emergence of new needs

Following a theoretical prediction in 1946 (Schwinger, 1946 ▸), synchrotron radiation was first observed with an electron-synchrotron accelerator in 1947 (Elder *et al.*, 1948 ▸). The first use of synchrotron radiation was reportedly a spectroscopic experiment in the vacuum ultraviolet region in 1963 (Madden & Codling, 1963 ▸). In the first-generation synchrotron radiation facilities, which were launched in the 1960s, accelerators dedicated for particle physics were used, *i.e.* synchrotron radiation not useful for elementary particle experiments was used parasitically.

In the 1980s, second-generation facilities dedicated for synchrotron radiation experiments were constructed in Japan, USA and Europe. Synchrotron radiation generated at a constant electron energy in storage rings was used. The FODO lattice (Courant *et al.*, 1952 ▸) (focusing and defocusing quadrupole magnets are placed alternately between bending magnets), which puts emphasis on beam convergence, was adopted, and its typical emittance was ∼100 nm rad. Improvements to the vacuum system and thermal countermeasures in the storage rings and the introduction of sextupole magnets led to increases in beam current and synchrotron radiation flux. In the 1990s, third-generation facilities were constructed with insertion devices installed in the straight sections. The double-bend achromatic (DBA) lattice (Chasman *et al.*, 1975 ▸), which had started to be adopted in the second generation, became the main type. The DBA places a focusing quadrupole magnet between two bending magnets in one cell, and its typical emittance was 1–10 nm rad. The reduced emittance and installation of an insertion device in the dispersionless straight section led to increases in synchrotron radiation brightness. Initially, two different types of rings were constructed in Japan, USA and Europe: small low-energy rings for vacuum ultraviolet and soft X-rays, and large high-energy rings for hard X-rays. Then, in the 2000s, medium-energy and medium-sized rings that can be used in a wide wavelength range were constructed worldwide, because top-up injection solved the problem of short Touschek lifetimes, and hard X-rays became available at low cost using in-vacuum undulators.

In the 2020s, the latest facilities are the fourth generation; these employ multi-bend achromatic (MBA) or hybrid multi-bend achromatic (HMBA) lattices (Tavares *et al.*, 2014 ▸; Farvacque *et al.*, 2013 ▸), with a typical emittance of ∼0.1 nm rad. The MBA (Tavares *et al.*, 2014 ▸) reduces the emittance by splitting the bending magnet to decrease the bending angle per magnet and suppress the dispersion. However, the MBA has a problem of small dynamic aperture. The HMBA (= MBA + DBA) (Farvacque *et al.*, 2013 ▸) solves this problem by efficiently making sextupole corrections in the DBA section with a large dispersion. The synchrotron radiation brightness is further increased by the ultralow emittance of the storage ring. The development of combined-function magnets (*i.e.* bending and quadrupole, quadrupole and sextupole), longitudinal-gradient bending magnets, small-diameter vacuum ducts with a non-evaporable getter (NEG) coating, and injection techniques that do not vibrate the stored beam was important to realize the fourth-generation facilities. With the increase in synchrotron radiation brightness, it is important to enhance the stability of not only the accelerator but also the entire facility.

The first generation demonstrated the usefulness of synchrotron radiation in materials and life sciences, its full-scale use started in the second generation, and its applications greatly expanded in the third generation. In the second generation, the focus was on flux-oriented research, but in the third generation, the focus broadened to brightness-oriented research. Furthermore, the fourth generation is expected to develop into research that utilizes spatial coherence. This trend is a natural consequence of the need to observe samples with higher spatial resolution. On the other hand, from a different evaluation perspective, the fourth generation may not be the highest-performance light source. With the introduction of highly sophisticated ultralow-emittance technology, some performance characteristics will be lower than those of the second and third generation. For the long-term sustainable development of synchrotron radiation science, we need not only a light source specialized for a specific performance but also a highly flexible light source that can generate synchrotron radiation best suited for each purpose of use. This would facilitate the trial and realization of researchers’ ideas. Accordingly, we propose the Hybrid Ring as a light source that enables new experimental techniques and enhances many existing ones.

## 2. Concept of the Hybrid Ring

**Figure 1:**
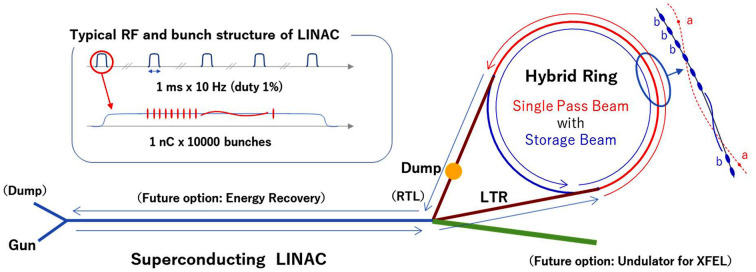
Concept of the Hybrid Ring with superconducting LINAC.

The Hybrid Ring is a variable light source with both versatility and advanced features that consists of a storage ring and a superconducting linac (Fig. 1 ▸). It is operated with the coexistence of the storage (SR) bunches characterized by the performance of the storage ring, and the single-pass (SP) bunches characterized by the performance of the superconducting linac. As the name suggests, the SP bunches pass through a part of the storage ring only once and are led to the beam dump. The 2.5 GeV storage ring at Photon Factory (PF), *i.e.* the institution of the authors, is operated not only with the usual mode, 280 bunches at 450 mA, but also with the hybrid mode, an isolated bunch at 30 mA + 130 bunches at 420 mA (450 mA in total). By association with the hybrid mode, the ring, which is based on a new concept of using two types of bunches, was named the Hybrid Ring. The SP bunches in the Hybrid Ring coexist with the SR bunches at a spatial distance. This is similar to the top-up injection where the injection beam is introduced in the presence of the stored beam. At the beamline, not only can the SR bunch and SP bunch beams be used selectively, but both can also be used simultaneously.

As for the lattice of the storage ring, we assume an intermediate lattice between the third and fourth generation. The ultralow emittance of the fourth generation and the narrow vacuum duct essential for its realization will be a major limitation to the use of vertically spread and polarized synchrotron radiation from a vertical wiggler and intense pulsed synchrotron radiation from large-charge isolated bunches, which are the features of the light sources of the PF. The former is suitable for deployment of X-ray optical elements in the horizontal plane, enabling, for example, the world’s widest-field highest-sensitivity X-ray phase-contrast imaging with a separated interferometer (Yoneyama *et al.*, 2013 ▸). The latter is useful, for example, for single-shot time-resolved measurements and nuclear resonant scattering measurements. For the SR bunches, it would be ideal to have the capability to enhance all the experimental techniques currently being implemented in the second- and third-generation facilities.

The superconducting linac is assumed to be a long-pulse type, which has already been used in X-ray free-electron laser (XFEL) facilities. For the SP bunches, the initial targets are an ultralow emittance of 0.1 nm rad with a charge of 1 nC and an ultrashort bunch length of 50 fs with 1 nC. The simultaneous realization of these three parameters and/or enhancement of each will be continued step-by-step. In addition to which parameter is given priority, there is a degree of freedom in the bunch structure within the long pulse. Utilization of this degree of freedom increases the flexibility as a light source. The superconducting linac will also be used for the multi-bucket top-up injection into the SR bunches.

Studies utilizing ultrashort bunches have been performed so far only with XFELs. An increase in the opportunity with the Hybrid Ring is expected to lead to significant progress in ultrafast-dynamics research.

The true value of the Hybrid Ring is demonstrated when two beams from the SR and SP bunches are used simultaneously. This is expected to enable new experimental methods using pump-and-probe and two-probe techniques by utilizing the respective properties of the two beams.

Superconducting linacs are often assumed to be expensive. However, for the long-pulse type used in XFELs, which has a pulse width of 1 ms and a duty cycle of 1%, the cost of construction and operation is about twice that of a normal-conducting linac. When the entire facility, including the storage ring, is considered, the increase in cost as a percentage is even smaller. Considering the new developments expected in synchrotron radiation science, the Hybrid Ring may be highly cost effective.

## 3. Light source and beamline of the Hybrid Ring

In the design study of the Hybrid Ring, priority will be given to the use of proven technologies, with emphasis on technical and economic feasibility. The study is in its early stage and is tentative, including the lattice type and electron energy of the ring. The results so far are described below.

### 3.1. Injector

The main parameters of the long-pulse superconducting linac are shown in Table 1 ▸. It is designed by optimizing the EuroXFEL (Decking *et al.*, 2020 ▸) linac (pulse width 1 ms and repetition rate 10 Hz = duty 1%). The accelerating gradient is 30 MV m^−1^ and the average current is 0.1 mA (10000 bunches/pulse). Eight nine-cell accelerating tubes of 1 m length are contained in a cryomodule. Since a maximum accelerating gradient of 35 MV m^−1^ is expected, it is possible to accelerate up to 3 GeV using 12 cryomodules with enough margin. Due to the long pulse width and large number of bunches in it, it will be necessary to install higher-order mode (HOM) couplers or dampers for the accelerating tube. As for the power supply, it needs to have the capacity to handle a peak power of 300 kW. Three 10 MW pulse klystrons, for example, will be able to drive the 12 cryomodules. For the refrigerator, the load due to heat inflow (static loss) is comparable with the radio frequency (RF) load (dynamic loss) because of the small duty cycle. Both are estimated to be ∼100 W at 2 K equivalent.

The characteristics of the SP bunch are mainly determined by the performance of the superconducting linac. In particular, the performance of the electron gun is important. As mentioned above, the initial targets are an emittance of 0.1 nm rad, a bunch length of 50 fs, and a charge of 1 nC, but we will continuously improve the performance of the SP bunch through research and development of the electron gun and other devices.

### 3.2. Ring

**Figure 2:**
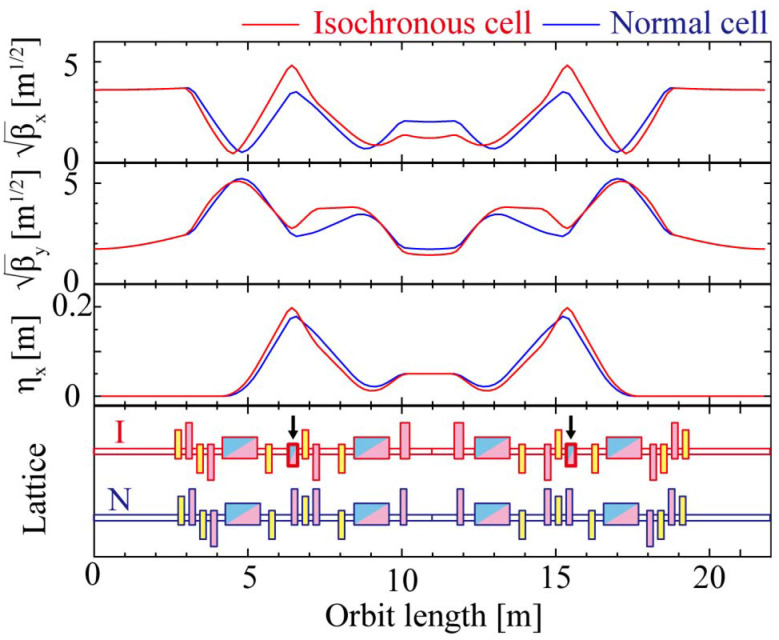
Optics and lattice of the Hybrid Ring. Blue, pink, and yellow boxes in the bottom figure denote bending, quadrupole, and sextupole magnets, respectively. Arrows indicate combined-function bending magnets with inverse polarity to make the beam optics isochronous.

The main parameters of the storage ring are shown in Table 2 ▸, and the lattice and optics are shown in Fig. 2 ▸. It is based on a double-DBA (DDBA) lattice (Nishimori *et al.*, 2019 ▸) (16 cells) of a new synchrotron radiation facility under construction in Japan. The lattice of the Hybrid Ring needs to balance two things: transporting the SP bunches while keeping its performance and maintaining enough dynamic aperture to ensure stable accumulation of the SR bunches.

The space-charge effect and coherent-synchrotron-radiation (CSR) effect should be considered as factors that may degrade the performance of SP bunches. For the space-charge effect, the data from the XFEL are helpful because it does not depend on the bending radius. It is less effective with increasing electron energy and does not become a serious problem at 3 GeV. The CSR effect occurs when a bunch of electrons passes through an arc (curved orbit) in bending magnets and the CSR generated from the tail of the bunch catches up with the head of the bunch through a string (shortcut), causing an intra-bunch energy transfer to decelerate electrons at the tail and accelerate electrons at the head. In order to reduce the CSR effect, the bending magnet should be shortened and the bending radius should be increased. In addition, by suppressing the dispersion in the arc sections, it is possible to suppress emittance growth and bunch lengthening. These measures to address the CSR effect are common to the design philosophy of the MBA lattice.

To realize the Hybrid Ring concept, specific design studies were conducted based on the DDBA lattice which is an intermediate of the third and fourth generation. The bunch lengthening caused by the original energy spread should be suppressed by isochronizing the cells through which the SP bunches pass, or eliminating the ‘R56’ element which represents the linear path-length deviation with respect to the energy difference. In the isochronous cell, combined-function bending magnets acting as a reverse bend are placed at the largest dispersion regions, and the principal bending magnets are strengthened to cancel them. On the other hand, since the momentum-compaction factor should be finite to make the whole ring work stably as a storage ring, only 10 of the 16 cells through which the SP bunches pass are set to isochronous.

**Figure 3:**
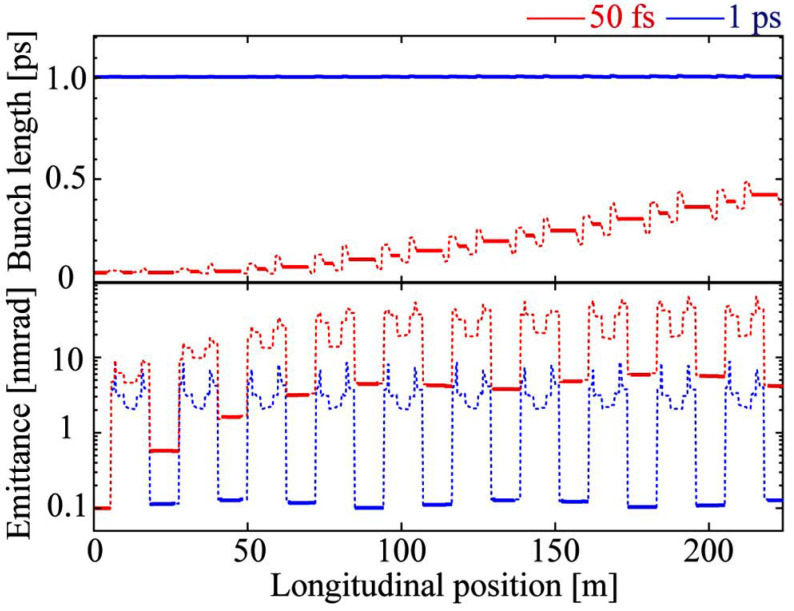
RMS bunch length and RMS emittance of the SP bunch with a charge of 1 nC in the isochronous cells.

**Figure 4:**
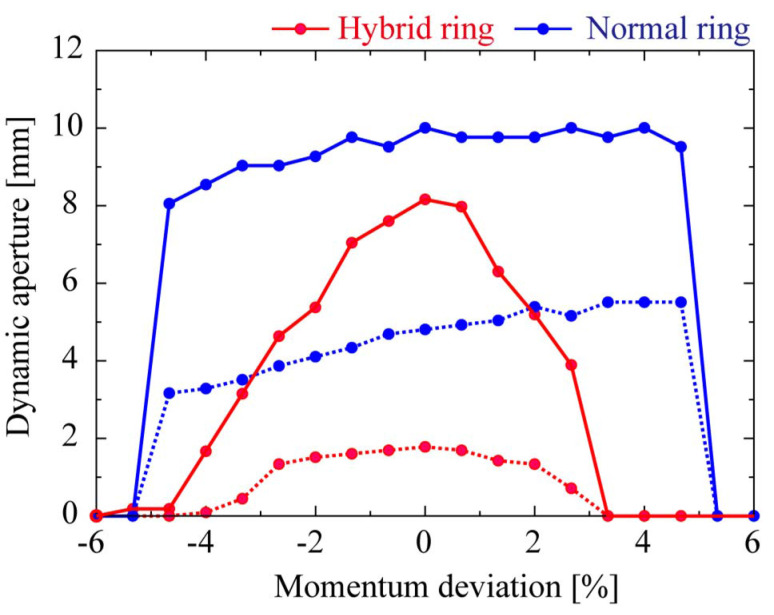
Dynamic apertures at the center of the long straight section. Solid and dashed lines indicate horizontal and vertical dynamic apertures, respectively.

The transportation of the SP bunches was calculated using the simulation code *Elegant* (Borland, 2000 ▸) and the results are shown in Fig. 3 ▸. In the isochronous cells, the bunch lengthening is sufficiently suppressed to maintain approximately 50 fs in the first few cells. In the case of a bunch length of 1 ps, an emittance of 0.1 nm rad is maintained. The average current of SP bunches is small (0.1 mA), but the charge is the same as that of SR bunches (1 nC), so the number of photons per SP bunch is not small. Compared with the case of slicing isolated bunches with a large charge (Zholents & Zolotorev, 1996 ▸), it is possible to use many orders of magnitude more photons at beamlines. The calculated dynamic apertures without errors are shown in Fig. 4 ▸. By optimizing the sextupole magnets, the horizontal aperture for injection has been so far recovered up to about 80% of the original 16-fold symmetric DDBA lattice (normal ring).

### 3.3. Beamline

At each beamline, beams from the SR and SP bunches will be used selectively or simultaneously. The beam can be selected by setting up a local orbit bump for each beamline. If two insertion devices are installed in the same straight section and the bump orbit is adjusted appropriately, it is possible to use two beams simultaneously with different optical axes. In order to use the two beams simultaneously, the optical axes of both should be adjusted independently. The trajectories of the SR and SP bunches are measured independently using the difference in the bunch repetition frequencies. The trajectory of the SP bunches is corrected without affecting the injection into the SR bunches, using pulsed multi-pole steering magnets, which can selectively kick the SP bunches with a finite horizontal amplitude.

**Figure 5:**
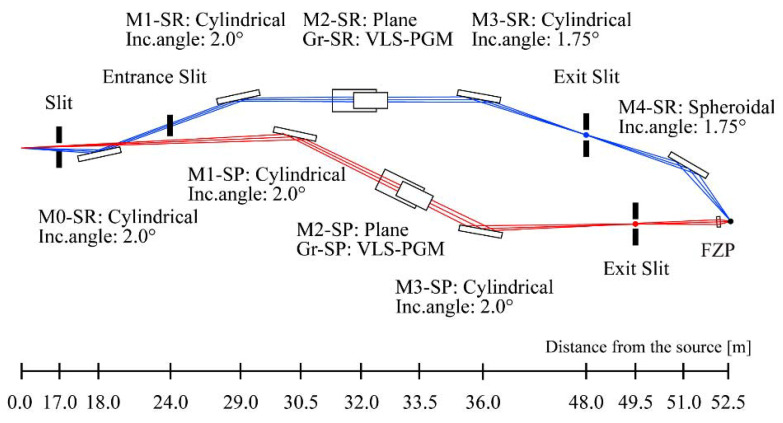
Example of the beamline design for soft X-ray and soft X-ray two-beam applications.

An example of the beamline that enables simultaneous use of the SR and SP beams is shown in Fig. 5 ▸. Here, both beams are assumed to be used in the soft X-ray region, and variable-included-angle optics with varied-line-spacing plane-grating monochromators (VLS-PGMs) (Amemiya & Ohta, 2004 ▸) are adopted. Although omitted in this paper, we are also studying the beamline designs for simultaneous use of two beams, one for soft X-rays and one for hard X-rays, and both for hard X-rays.

In the case of the beamline shown in Fig. 5 ▸, the SR and SP beams are emitted with an azimuthal difference of 1 mrad and focused to 10 µm and 50 nm, respectively, at the sample position at a distance of about 50 m. The optics for the SR beam are configured to be applicable to a variety of experimental methods by making full use of multiple mirrors. The M0-SR mirror separates the optical path from the SP beam and focuses the beam to the entrance slit. The M3-SR mirror, which has a striped coating, eliminates harmonics and allows for a wide range of energy selection. The optics for the SP beam, on the other hand, have a simple configuration that emphasizes reducing the flux loss. Since the flux of the SP beam is small, there is no heat load problem. The M3-SP mirror processes harmonics.

When we consider the simultaneous use of two beams, adjustment of the focusing position is important. Targeting the focal point of the SR beam, the focusing position of the SP beam is coarsely adjusted by the output angle of the M3-SP mirror, and then finely tuned by the position of the Fresnel zone plate (FZP). To prevent unintended relative positional fluctuations of the two beams, the exit slits, the M4-SR mirror, and the FZP are mounted on a single robust frame. Both beams are expected to be operated in several typical bunch structures, and a measurement system with sufficient time resolution, especially for the SP beam, is necessary for their effective utilization.

### 3.4. Issues

The main issues for the realization of the Hybrid Ring are to secure the dynamic aperture as a storage ring, to take measures against beam loss from the viewpoint of radiation safety and device protection, and to study the details of the hardware.

For the dynamic aperture, we plan to further optimize the sextupole magnets (non-linear optics) and to study the effects of magnetic errors from the ideal state, the effects of local changes due to the control of pulsed multi-pole steering magnets, and the effects of the injection and the extraction. Although the average current of the SP bunches is low, the effect of beam loss is enormous because the long pulse coming at 10 Hz contains 10 to 100 times the charge of the SR bunches for the whole ring. When beam loss is observed, it is essential to stop the linac instantaneously. We are working on developing faster interlocks. We are also working on remote control of the beamline to restrict human access to the specific areas where the radiation level increases during beam loss. In addition, it is necessary to proceed with detailed studies of the hardware for the ring and the beamline, including the performance of each component and the avoidance of interference between the components. For the injector, no new technology is required, but, since the goal is to achieve higher current values than have been realized to date, it is important to improve the performance of each element and to test the operation of the system as a whole.

## 4. Science cases for the Hybrid Ring

The Hybrid Ring will enable unique experiments to be performed by simultaneous use of two beams, in addition to research with various experimental techniques utilizing the SR beam and ultrafast-dynamics research using the ultrashort pulse of the SP beam. Here, we present the science case where the SR and SP beams are used as probes, and the case where the sample is pumped with the SP beam and then the temporal changes are probed with the SR beam.

### 4.1. Example of SP and SR probes

**Figure 6:**
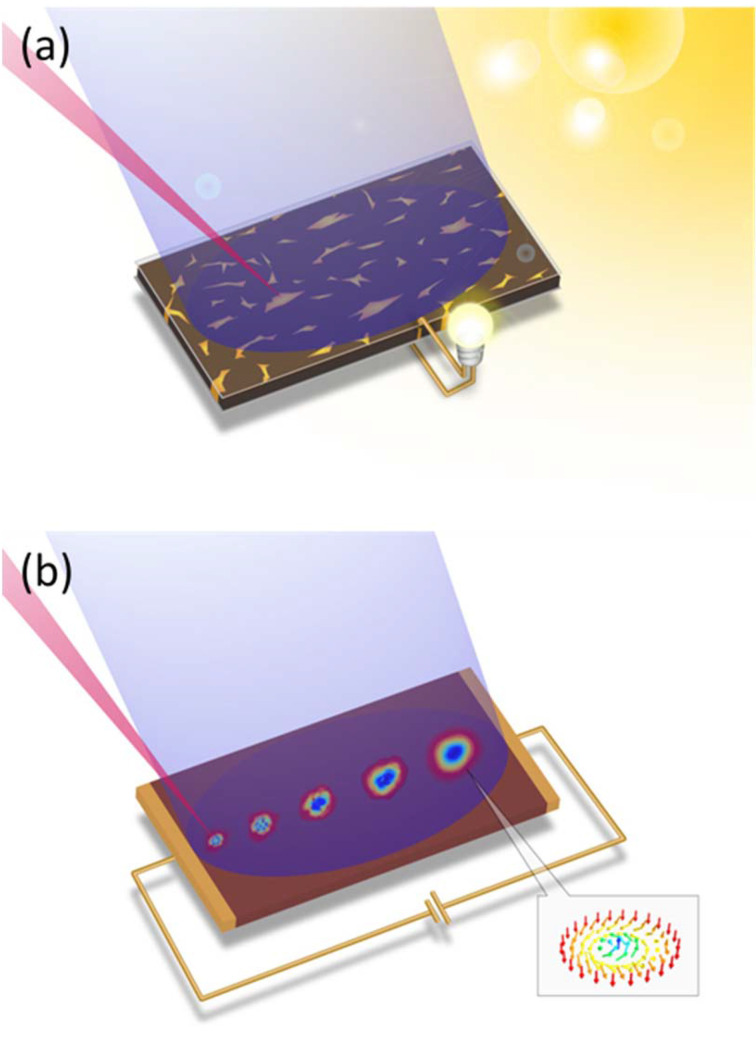
Examples of two-beam application. (*a*) Two X-ray probes for a solar cell and (*b*) X-ray pump and X-ray probe for a spintronics device. In both cases, the relative position of the SP and SR beams is adjustable.

Fig. 6 ▸(*a*) shows the observation of the photochemical reaction in a solar cell as an example of using the two beams as probes. Photochemical reactions are expected to be applied to artificial photosynthesis, solar cells, photocatalysts, and so on (Fujishima & Honda, 1972 ▸; Kojima *et al.*, 2009 ▸; Tachibana *et al.*, 2012 ▸). In this research field, dynamic information on the structure and electronic state of small regions that play an important role in reactions is required. The desired information is obtained by continuously observing gradual temporal changes (>1 ms) of the entire sample in operation using wide-field imaging of the SR beam to identify regions of interest (*e.g.* grain boundaries and crystal planes), and by observing ultrafast phenomena (*e.g.* photocarrier dynamics, redox reactions, and local structural changes) in those regions using the nano-focus (<100 nm) and ultrashort-pulse (<1 ps) properties of the SP beam. Thus, by using the two beams as probes, it is possible to follow the behavior of the sample in a wide spatial-temporal hierarchical structure.

### 4.2. Example of SP pump and SR probe

Fig. 6 ▸(*b*) shows the observation of the generation process of a nano-scale magnetic structure as an example of using the SP beam for pumping and the SR beam for probing. The technique of controlling nano-scale spin texture is expected to be applied to highly integrated next-generation magnetic storage media (Mühlbauer *et al.*, 2009 ▸; Yu *et al.*, 2010 ▸; Nagaosa & Tokura, 2013 ▸). It has been theoretically predicted that skyrmions, which are known for their topologically protected spin structure, can be introduced into magnetic materials by exciting them with nano-focused ultrashort pulses of vortex light (Fujita & Sato, 2017 ▸). The SP beam, focused to a small size (<100 nm) comparable with that of skyrmions with a shorter pulse than their relaxation time (1 ps), is used to write skyrmions, and the SR beam is used to observe the temporal generation process of the skyrmions with picosecond time resolution to gain insight into the generation and control of data carriers. Thus, by utilizing the performance of the SP beam as a pumping light to create a unique state, it is possible to reveal the subsequent change of state using the SR beam as a probe.

## 5. Development into a synchrotron radiation complex

This paper has proposed the Hybrid Ring with a superconducting linac as an injector as a light source to enable new experimental techniques and enhance many existing ones (Fig. 1 ▸). The Hybrid Ring is a flexible and extendable light source capable of generating synchrotron radiation suited for each purpose of use. The performance of SP bunches can be improved step-by-step by research and development of the linac, especially the electron gun. In addition, the electron energy can be increased by reinforcing the linac. When the average current or electron energy is increased, energy recovery may be required to safely dump the SP beam, which would also help reduce the power consumption. If the upper limit of the electron energy that can be accelerated by the injector is expanded, simultaneous injection into two rings, one with low energy and the other with high energy, and XFELs up to the hard X-ray region may become feasible. Thus, the extendability of the Hybrid Ring will allow it to be developed into a synchrotron radiation complex that contributes to a wide variety of research.

## Figures and Tables

**Table 1:** Tentative parameters of superconducting LINAC

Parameter	Value
Electron beam	
Energy (GeV)	3
Averaged current (mA)	0.1
Bunch charge (nC)	1
Normalized emittance (mm mrad)	0.6
Natural emittance (nm rad)	0.1
Bunch length (fs)	50
Energy spread (%)	0.50

Superconducting LINAC	
RF frequency (GHz)	1.3
Accelerating gradient (MV m^−1^)	30
Number of nine-cell accelerating tubes	96
Number of cryomodules	12
Macro pulse repetition rate (Hz)	10
RF pulse length (ms)	1
RF flat-top length (ms)	0.6
Bunch numbers/pulse	10000
Bunch repetition frequency within pulse (MHz)	18
RF load (2 K) for cryosystem (W/module)	8
Static loss (2 K) for cryosystem (W/module)	8
Total capacity (2 K) of cryosystem (W)	200

**Table 2:** Tentative parameters of the Hybrid Ring

Parameter	Normal ring	Isochronous	Hybrid ring
Energy (GeV)	3	3	3
Circumference (m)	350	350	350
Lattice	DDBA	DDBA	DDBA
Number of normal cell	16	0	6
Number of isochronous cell	0	16	10
RF voltage (MV)	3.6	–	3.6
RF frequency (MHz)	500.0	–	500.0
Bucket height (%)	4.70	–	7.30
Energy loss (MeV/turn)	0.62	0.83	0.75
Momentum compaction	4.26 × 10^−4^	0.00	1.59 × 10^−4^
Betatron tune, ν_*x*_/ν_*y*_	28.17/9.23	28.17/9.23	28.17/9.23
Damping time, *x*/*y*/*z* (ms)	8.12/1.12/6.93	3.23/8.43/21.7	4.17/9.30/12.1
Storage current (mA)	500	–	500
Natural emittance (nm rad)	1.15	0.55	0.66
Energy spread	8.42 × 10^−4^	1.80 × 10^−3^	1.26 × 10^−3^
Natural bunch length (ps)	9.64	–	8.84
